# Determining Trendelenburg test validity and reliability using 3-dimensional motion analysis and muscle dynamometry

**DOI:** 10.1186/s12998-020-00344-3

**Published:** 2020-10-19

**Authors:** Luke McCarney, Alexander Andrews, Phoebe Henry, Azharuddin Fazalbhoy, Isaac Selva Raj, Noel Lythgo, Julie C Kendall

**Affiliations:** 1 Osteopathy, School of Health and Biomedical Sciences, RMIT University, Bundoora, VIC 3083 Australia; 2 Chiropractic, School of Health and Biomedical Sciences, RMIT University, Bundoora, VIC 3083 Australia; 3 Exercise and Sports Sciences, School of Health and Biomedical Sciences, RMIT University, Bundoora, VIC 3083 Australia

**Keywords:** Hip, Trendelenburg test, Muscle strength, Biomechanics, Vicon

## Abstract

**Background:**

The hip abductor muscle group stabilises the pelvis during gait to prevent excessive pelvic drop. Hip abductor weakness has been linked to musculoskeletal conditions such as chronic low-back pain. As such, it is important that practitioners can correctly diagnose hip abductor weakness in a clinical setting. Although the Trendelenburg test is commonly used by practitioners, the validity of this test to assess hip abductor weakness in the absence of musculoskeletal injury remains questionable. The aim of this study was to determine the validity of the Trendelenburg test, as observed by a practitioner, to assess frontal plane pelvic motion and hip abductor strength in a population without intra-articular hip disorders.

**Methods:**

This study was performed between June 14th and October 16th 2019. Eighteen participants were recruited for this study. Peak normalised isometric and isokinetic hip abductor torque were measured bilaterally (*n* = 36) using the Biodex System 4 isokinetic dynamometer. Each participant performed the Trendelenburg test bilaterally (*n* = 36) while a graduate year chiropractic practitioner assessed for a “positive” or “negative” sign. The test was simultaneously recorded using Vicon 3-Dimensional motion capture to measure frontal plane pelvic motion and elevation. Correlation analyses were performed between the measures of peak hip abductor torque and pelvic motion to determine if any relationship existed. Agreement between the practitioner and 3-Dimensional analysis was calculated using the kappa (κ) statistic.

**Results:**

Weak, non-significant correlations were found between hip abductor strength and pelvic motion before outlier removal. Significant (*p* < 0.05) yet weak correlations were found after outlier removal, except for isometric hip abductor strength. Weak agreement was found between the chiropractic practitioner and 3-Dimesnional analysis for the Trendelenburg test assessment (κ = 0.22–0.25).

**Conclusions:**

This study found no significant relationship between normalised peak isometric and isokinetic hip abductor torque and frontal plane pelvic motion during the Trendelenburg test in a healthy young adult population. There was also poor agreement between the practitioner and pelvic motion assessments. Caution should be used when using this test, in the absence of intra-articular hip pathology, to assesses hip abductor weakness. Before any definitive conclusion can be made, studies with a larger sample size should be performed.

## Background

A primary function of the hip abductor muscle group is to stabilise the pelvis during gait and prevent excessive frontal plane pelvic drop, sometimes referred to as pelvic list [1–3]. Weakness of the hip abductors has been linked to musculoskeletal conditions such as chronic low-back pain [4–6], patellofemoral and anterior knee pain [7–9], gluteal tendinopathy [10, 11] and osteoarthritis of the hip joint [11–13]. As such, it is important that practitioners can correctly diagnose hip abductor weakness in a clinical setting. Current techniques used for diagnosis include manual muscle testing [4], handheld dynamometry [14, 15] and functional orthopaedic tests such as the Trendelenburg test [16, 17].

A common approach used to assess hip abductor strength involves manual muscle testing where a practitioner grades the strength of the hip abductors (from 0 to 5) by physically resisting a patient’s hip abductor movement [4]. A limitation of this approach is that it relies on the practitioner to subjectively grade or rate the patient’s strength, and it is difficult to objectively differentiate between the higher grades [4]. An objective approach involves the use of handheld strength dynamometers or fixed station dynamometers (e.g. Biodex) to measure hip abductor strength [14, 15, 17]. If performed correctly, these devices can accurately assess hip abductor strength [15, 18–20]. Fixed station isokinetic dynamometry is considered the “gold standard” for the assessment of muscle strength [19, 20]. A high level of training and skill, however, is required for these systems. Furthermore, systems such as the Biodex are prohibitively expensive and not portable. Another common approach involves the use of an orthopaedic assessment known as the Trendelenburg test.

The Trendelenburg test, developed in 1897 by Frederick Trendelenburg, is currently used to screen for hip osteoarthritis and weakness in the hip abductor muscle group [21–23]. There are several variations of this test; however, a commonly described method used by practitioners requires a patient to stand on one leg, flex the hip on the non-stance leg side to approximately 30°, followed by elevating the pelvis on the non-stance leg side “as high as possible” with this position maintained for a period of 30 s [22]. Failure to keep the pelvis maximally elevated for the 30 s period is deemed to indicate weakness in the hip abductors and is classified as a “positive” Trendelenburg test or sign [18].

Although the Trendelenburg test is commonly used by practitioners, its validity to assess hip abductor weakness, particularly in the absence of musculoskeletal injury, remains questionable [18]. For example, a study where hip abductor strength in a group of healthy male adults was significantly reduced through a nerve block technique found no significant effect on pelvic drop during the Trendelenburg test [18]. A further study by this group, where hip abductor strength was significantly increased through hip abductor resistance training in a group of adults with chronic low-back pain, also found no significant effect on pelvic drop during the Trendelenburg test [6]. It should be noted that these studies did not have a practitioner assess the Trendelenburg test as either “positive” or “negative”, nor was the frontal plane vertical displacement of the pelvis investigated. These factors are important as they provide clinical relevance and accurate quantitative assessment of pelvic drop. As such, clinical accuracy was not investigated in these studies. Previous work has investigated inter-rater reliability of the Trendelenburg test [5] but it only involved adults (average age = 37 years) with low back pain [5]. A systematic review found poor-to-good sensitivity and good-to-excellent specificity in adult populations with gluteal tendinopathy and gluteus medius strains [16]. The aim of this study was to determine the validity of the Trendelenburg test, as observed by a practitioner, to assess frontal plane pelvic motion and hip abductor strength in a population without intra-articular hip disorders. A secondary aim was to investigate the agreement between a chiropractic practitioner’s subjective assessment of the Trendelenburg test in comparison to a highly accurate, quantitative 3-dimensional measurement of frontal plane pelvic motion during the test.

## Methods

This study and the relevant data collection were performed between June 14th and October 16th 2019. A sample of convenience was recruited for this study. Participants were excluded if there was any previous or current hip pathology that would prevent them from participating in the study. Participants were also excluded from the study if they had any current musculoskeletal injuries and neurological conditions that may affect balance and posture. For example, people with conditions such as cerebral palsy and fibromyalgia were excluded from the study. Prior to performing the test, participants received an explanation of how to perform the manoeuvre, including all risks and benefits, and read a plain language statement. Participants were familiarised with the Trendelenburg test before assessment and practiced the manoeuvre until they were able to satisfactorily demonstrate to the practitioner they were correctly performing the test. The Trendelenburg assessment was conducted by a first-year practicing and licensed chiropractor (12 months) with 2 years of student clinical placement experience. The participants provided written informed consent. This study was approved by the RMIT Science, Engineering and Health College Human Ethics Advisory Network (CHEAN).

### Experimental set-up and protocol

#### 3-dimensional motion capture

Frontal plane angular pelvic position was captured during the Trendelenburg test. These data were captured using six Vicon (Oxford Metrics, UK) infrared motion capture cameras sampling at 120 Hz. Fifteen passive spherical reflective markers (14 mm diameter) were placed on known anatomical landmarks on the lower extremities (Vicon Plug-in-Gait marker set) using non-allergenic double-sided tape and further secured with micropore tape. Anatomical landmarks were first identified (standing position) by an eyeliner pen. Markers were then placed over the anterior superior iliac spines (ASIS), on the sacrum at the mid-way point between the posterior superior iliac spines (PSIS), on the lateral epicondyles of the knees in line with the joint axis and on the lateral thigh along the line from the knee marker to greater trochanter. The ankle markers were placed on the lateral malleolus along an imaginary line that passes through the transmalleolar axis, a lower leg marker was placed on the lateral aspect of the lower leg along the line between the knee and ankle markers. Toe markers were placed over the second metatarsal head, on the mid-foot side of the equinus break between fore-foot and mid-foot, and heel markers were placed on the calcaneus at the same height above the plantar surface of the foot as the toe marker.

Anthropometric measurements were taken and used by the Vicon Nexus software (version 2.7.1, Oxford, UK) to create a lower extremity biomechanical model of the participant in order to extract joint angular data. Measurements recorded were height (mm), mass (kg), inter anterior superior iliac spine distance (mm), leg length (mm), knee width (mm) and ankle width (mm). Research quality callipers from an anthropometric measuring set (Mentone Educational, Australia) were used to measure ASIS, knee and ankle widths. The anthropometric measuring tape from the measuring set was used to measure leg length (ASIS to medial malleolus) as described by the plug-in gait model. An electronic weight scale measured mass. An analogue stadiometer was used to measure height.

The global reference frame (5 marker wand) was positioned in the middle of the laboratory on the corner of an in-ground force plate. With this setup, the y-axis ran length ways, the x-axis sideways and z-axis vertically to the laboratory with each axis perpendicular to the others; an orthogonal axis system. The spirit levels on the “five-marker” wand were adjusted to ensure the global reference frame was level with the ground. Participants were positioned in the centre of the laboratory facing an end wall. Their body was positioned so that any forward movement was along the y-axis, sideward movement along the x-axis and vertical movement along the z-axis (laboratory-based global axis system).

#### Dynamometry

**Fig. 1:**
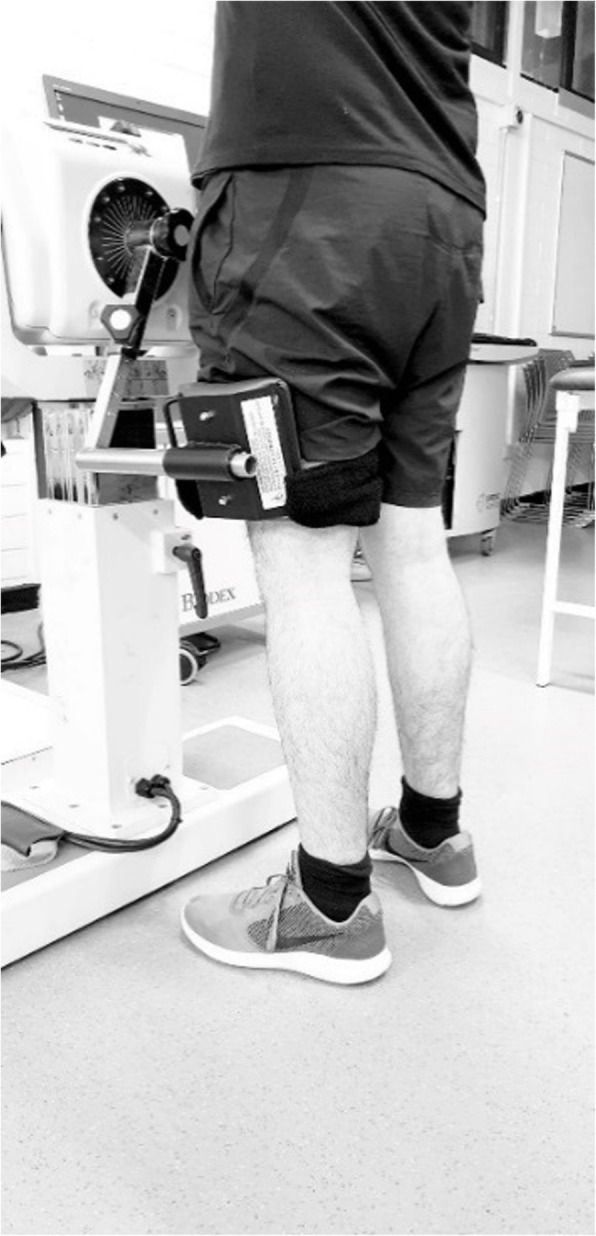
Biodex setup for the assessment of hip abductor strength

Before assessment, participants were familiarised with the static and dynamic Biodex hip abduction tests. This involved instructing the participant and sub-maximal performances until they were comfortable with the test and were considered ready for assessment by the practitioner. Following the familiarisation period, hip abductor torque (Nm) was recorded by the Biodex System 4 (Biodex, New York, US) isokinetic muscle dynamometer. Hip abductor torque was recorded in a standing position. The shaft of the dynamometer was aligned to the hip joint axis of rotation, approximately at the level of the greater trochanter. The attachment for the Biodex was placed on the lateral aspect of the side being tested, slightly superior to the popliteal fossa (Fig. 1) and was then fixed in place by strapping [24].

For the dynamic hip abductor test, the Biodex program was set to the isokinetic protocol. The range of hip frontal plane motion, that is hip abduction, was defined before each test. The range of hip abduction was from a neutral standing position (anatomical position) to the point of maximal voluntary abduction by a participant. Two discrete sets of five repetitions were performed on each side at an angular velocity of thirty degrees per second (30°·s^− 1^) [25–27] with a 30 s rest period between each set, as a 1:3 work to rest ratio is a common protocol when assessing isokinetic muscular strength [28]. The participant was asked to abduct their leg as hard and fast as possible within the range of hip abduction previously recorded. The participant had to remain upright and limit the recruitment of other muscle groups to assist the action. An investigator stood in front and to the side of the participant to prevent them from using trunk sway and to also provide support.

For static testing, the Biodex was set to the isometric mode. Five repetitions of 5 s contractions were performed on each leg with a 20 s rest period between each repetition [29]. The participants were instructed to abduct their leg against the Biodex resistance “as hard as possible and maintain the contraction for five seconds”. The participants were asked to remain upright and not use other muscle groups to assist the action.

#### Trendelenburg test

**Fig. 2:**
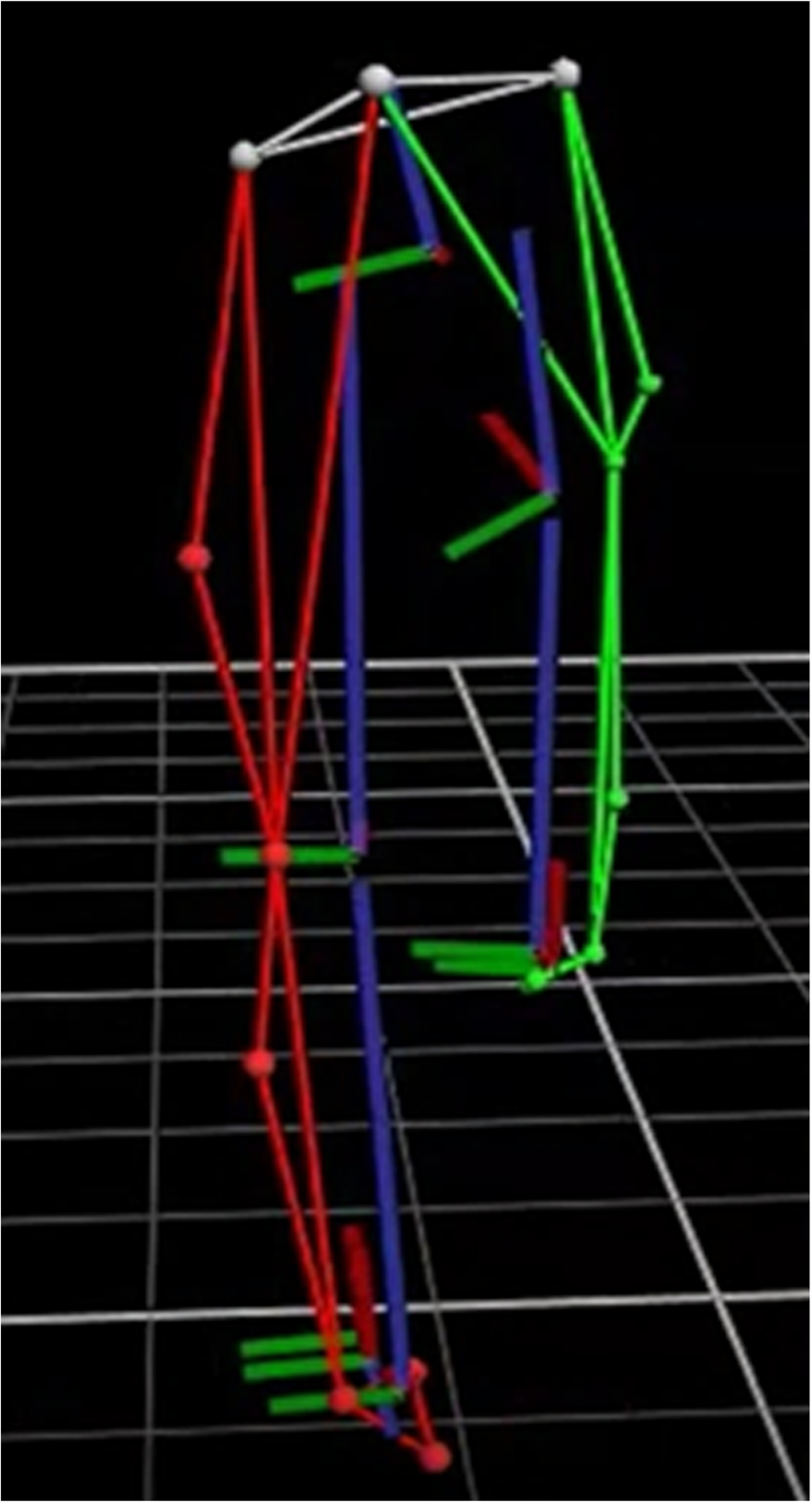
Performing the Trendelenburg test. The image shows the 3-Dimensional motion capture by the Vicon system as a particiapant perfroms the Trendelenburg test (posterior view). The right leg is identified by the colour “green” and the left leg is identified by the colour “red”. In this figure the frontal plane pelvic angle on the right side of the pelvis is approximately 25°

The participants were instructed to stand with their arms held across the chest. They then raised one leg in the air to approximately thirty degrees of hip flexion (sagittal plane); approximately where the first toe of the non-stance leg is aligned with the medial malleolus of the stance leg. The participant was then asked to raise the pelvis of their non-stance leg as high as possible and maintain this position for 30 s (Fig. 2). Participants could steady themselves by lightly touching a support bar (mid-trunk height) positioned to the side with one finger. The examiner could not provide support as it occluded some markers from the view of the 3D cameras. After 30 s the participant lowered their leg and the test was repeated on the opposite side. The test was performed in accordance with the instructions described by Hardcastle and Nade [22].

While performing the test, the practitioner observed the participant and determined whether the test was a “positive” or “negative” sign. A “positive” sign was considered to be any drop of the pelvis observed on the non-stance side, or shift of the trunk towards the stance side so as to compensate for hip abductor weakness [22]. A “negative” sign was when no drop in pelvic height or trunk lean to the side of the stance leg was observed [22]. While the participant performed the test, Vicon captured the movement of the lower extremity to objectively measure the changes in pelvic height and frontal plane pelvic angular motion.

### Data collection and analysis

Peak isometric and isokinetic hip abductor torque (Nm) data were collected from each repetition performed on the Biodex. Outliers were removed for each participant using inter-quartile range (IQR) and the average (local mean) peak isometric and isokinetic hip abductor torque was recorded (normalized to body mass). Normalizing torque to body mass (Nm·kg^− 1^) is typically used to analyse strength data [30]. These local mean data were then used to calculate global descriptive statistics (Mean ± SD) and for all analyses.

The frontal plane angular position (°) and vertical displacement (mm) of the pelvis were captured by the Vicon system during the Trendelenburg test. These data were filtered with a Woltring filtering routine (MSE = 20) which is commonly used in motion analysis [31]**.** These data were extracted from both sides of the pelvis (on the non-stance side) during the Trendelenburg test. The maximum and minimum pelvic angular positions (°) were used to calculate the angular displacement of the pelvis in the frontal plane during the Trendelenburg test; defined in this paper as pelvic drop (P_Drop_). The maximum and minimum vertical displacements of the pelvis were used to calculate the vertical displacement of the pelvis in the frontal plane during the Trendelenburg test; defined in this paper as pelvic displacement (P_Disp_). The marker on the ASIS of the non-stance leg was the point used to determine P_Disp_. The marker’s maximum and minimum vertical position was measured relative to the laboratory using the global z-axis described above. Participants could correct an imbalance without failing or having to restart the test. If a minimum or maximum pelvic position occurred during a period of imbalance, these data were not extracted during processing. Minimum and maximum data were only extracted from periods where the test was performed correctly.

Descriptive statistics and measures of normality were calculated for each data set. Pearson’s correlation analyses were performed to investigate the relationship between peak isometric and isokinetic hip abductor torque and the measures of P_Drop_ and P_Disp_. If a significant correlation was found, a linear regression was performed on the data sets. If a non-significant correlation was found, scatter plot analyses were performed. Outliers were removed from the plot and pool of data using visual inspection combined with IQR analysis. These data were removed on the basis that they were considered to be extreme and not truly representative of the population [32]. Pearson’s correlation coefficients for each data set were recalculated with the outliers removed to observe the effect of outlier removal. Statistical analyses were performed using GraphPad Prism (8.1.2, California, US).

Further analysis involved categorizing participants into groups; i.e. Trendelenburg “positive” or Trendelenburg “negative”. This was based on the assessment of the chiropractic practitioner as defined by Hardcastle and Nade (1985). Descriptive statistics (Mean ± SD) and measures of normality were calculated for the P_Drop_ and P_Disp_ group data and listed in Table 2. Group data were then compared by using an independent t-test.

P_Drop_ and P_Disp_ data were used to investigate a “clinically significant change” (CSC), sometimes referred to as the “minimal clinically important difference” (MCID) [33]. This statistic is commonly used to identify change beyond an expected measurement error. For this paper, a one-tailed right 99% confidence interval (CI) was calculated for the MCID [34, 35]. This statistic was used to determine a cut-off score for the P_Drop_ and P_Disp_ data that indicates a “positive” or “negative” Trendelenburg result based on the data extracted from the Vicon analysis. That is, if a participant’s P_Drop_ or P_Disp_ data fell beyond the 99% CI, it was considered a “positive” Trendelenburg test, and anything below a “negative” test outcome. These results were then compared, through the kappa (κ) statistic, to the results given by the chiropractic practitioner to calculate a measure of agreeance [36]. This statistic is a chance-weighted measure of agreement used to assess the level of agreement; that is, it removes the effects of chance agreement.

## Results

Eighteen volunteer healthy young adults (8 male, 10 female, age = 24.6 ± 4.9 years, height = 1.75 ± 0.1 m, mass = 73.4 ± 10.3 kg) participated in this study. The group’s average BMI was 23.9 (SD = 2.57) with a range of 20.4 to 27.6. The average BMI falls well below an obese level. A power analysis was not conducted because the aim the study was to evaluate the use of a clinical test to identify pelvic motion as opposed to detecting a “treatment effect” in a population based on a sample drawn from the population [35].

In this study, both pelvic sides of each participant were assessed (*n* = 36). All data sets were found to exhibit normality with skewness and kurtosis measures ranging from − 0.86 to 1.49 [37]. Descriptive statistics are listed in Table 1.

**Table 1:** Descriptive statistics for P_Drop_, P_Disp_, peak isometric and isokinetic torque. The table shows that the participants generated greater torque during the isokinetic testing. On average, pelvic drop was 4.6° and pelvic vertical displacement was 17 mm

Outcome Measure	Mean ± SD	n
Peak Isometric Torque (Nm**·**kg^− 1^)	0.65 ± 0.16	36
Peak Isokinetic Torque (Nm**·**kg^− 1^)	0.71 ± 0.19	36
P_Drop_ (°)	4.6 ± 1.7	36
P_Disp_ (mm)	17 ± 8	36

**Fig. 3:**
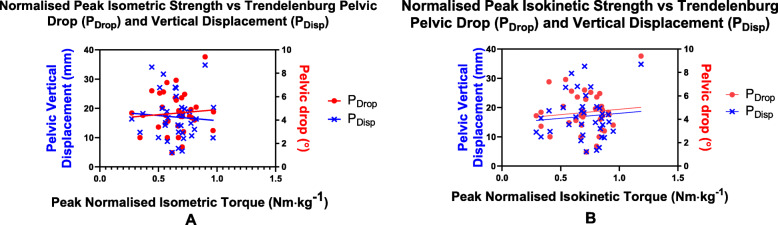
**a** Scatter plots of normalised peak isometric hip abductor torque and P_Drop_ and P_Disp_ (*n* = 36). **b** Scatter plots of normalised peak isokinetic hip abductor torque and P_Drop_ and P_Disp_. (*n* = 36)

Low to weak and non-significant correlations were found between peak isometric torque and P_Drop_ (*r* = 0.08, *p* = 0.65; Fig. 3a) and P_Disp_ (*r* = − 0.07, *p* = 0.68; Fig. 3a). Low to weak and non-significant correlations were also found between the peak isokinetic torque and P_Drop_ (*r* = 0.11, *p* = 0.54; Fig. 3b) and P_Disp_ (*r* = 0.09, *p* = 0.61; Fig. 3b).

**Fig. 4:**
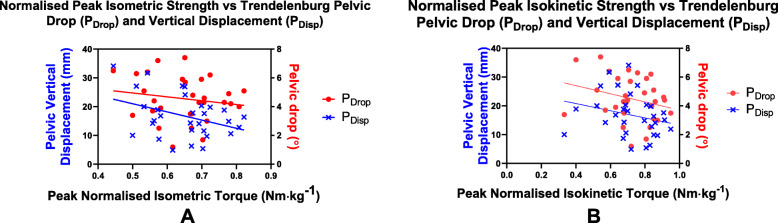
**a** Scatter plots of normalized peak isometric peak hip abductor torque and P_Drop_ and P_Disp_ with outliers removed (*n* = 30). **b** Scatter plots of normalized peak isokinetic hip abductor torque and P_Drop_ and P_Disp_ with outliers removed (*n* = 31)

Six outliers were removed from the peak isometric torque data set for further analysis (*n* = 30). After removal of the outliers, a low to weak and non-significant correlation was found between peak isometric torque and P_Drop_ (*p* = 0.38, Pearson’s *r* = − 0.17; Fig. 4a). A significant yet weak negative correlation was found between this torque and P_Disp_ (*r* = − 0.37, *p* = 0.046; Fig. 4a) data.

Five outliers were removed from the isokinetic data for further analysis (*n* = 31). After removal of the five outliers (*n* = 31), significant but low negative correlations were found between peak isokinetic torque and P_Drop_ (*r* = − 0.38, *p* = 0.04; Fig. 4b) and P_Disp_ (*r* = − 0.41, *p* = 0.03; Fig. 4b).

**Fig. 5:**
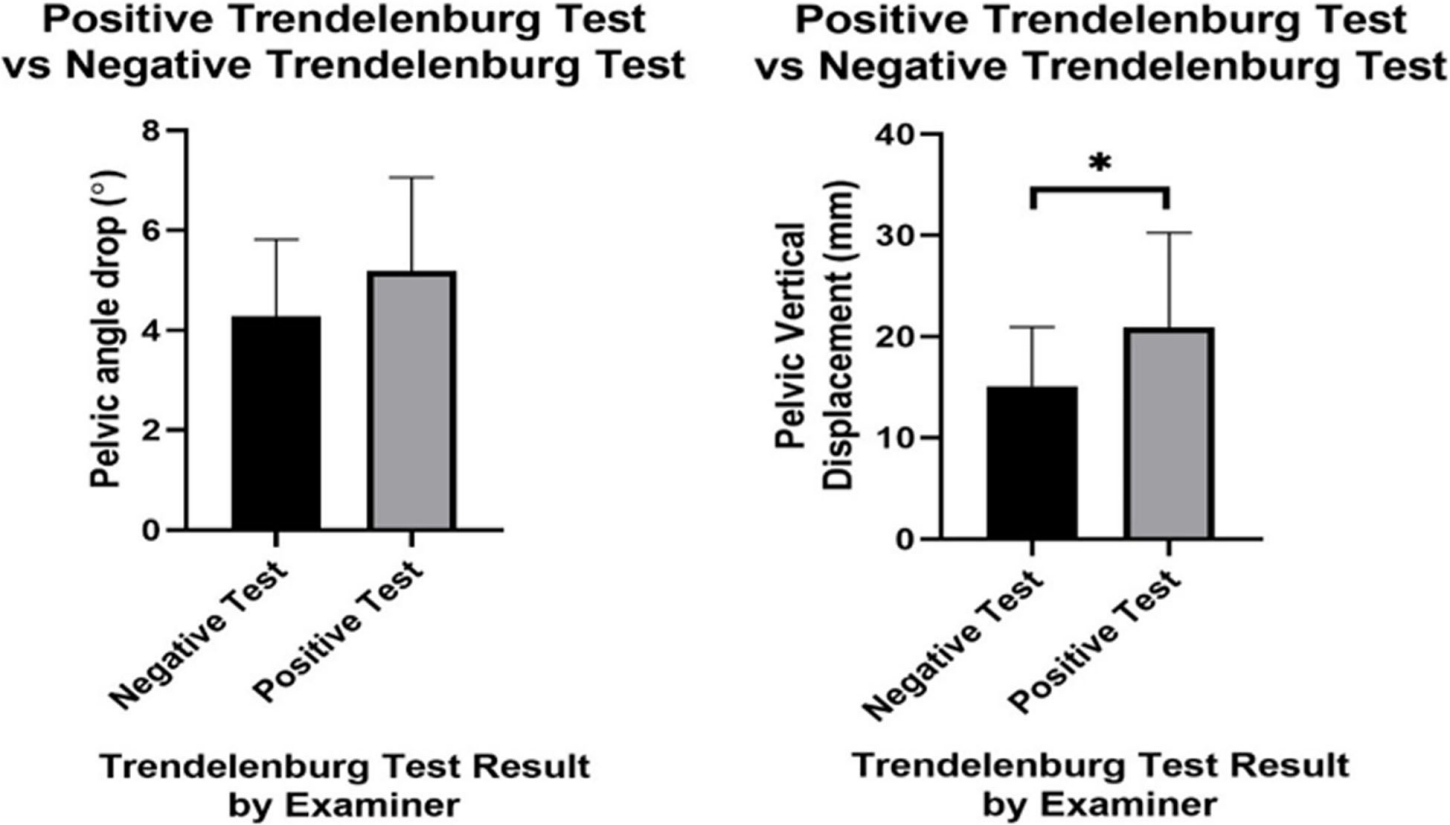
Plots of P_Drop_ and P_Disp_ “positive” and “negative” group. *p = 0.03

Of the 36 Trendelenburg tests performed, the chiropractic practitioner determined that 12 assessments showed a “positive” Trendelenburg sign, and the remaining 24 were a “negative” sign. For the “positive” Trendelenburg group the average P_Drop_ (mean ± SD) was 5.2° ± 1.9° and the average P_Disp_ was 15.1 ± 5.9 mm. For the “negative” Trendelenburg group the average P_Drop_ was 4.3° ± 1.7° and P_Disp_ was 21 ± 9.3 mm. Descriptive statistics are listed in Table 2. All data sets, before removal of any outliers (*n* = 36), were found to exhibit normality. No significant difference was found between the “positive” and “negative” groups for P_Drop_ (Fig. 5) where the average group difference was 0.9°. A significant difference was found between the “positive” and “negative” groups for P_Disp_ (*p* = 0.03; Fig. 5) with an average difference of 6 mm.

**Table 2:** Descriptive statistics for P_Drop_ and P_Disp_ for the “positive” and “negative” groups

Group (+/−) and Outcome Measure	Mean ± SD	n
P_Drop_ (°)^+^	5.2 ± 1.9	12
P_Disp_ (mm)^+^	21 ± 9	12
P_Drop_ (°)^−^	4.3 ± 1.7	24
P_Disp_ (mm)^−^	15 ± 6	24

The MCIDs for P_Drop_ and P_Disp_ (*n* = 36) were 5.3° and 20 mm respectively. Upon re-examination of the data utilizing these cut-off scores, ten P_Drop_ and nine P_Disp_ assessments were classified as a “positive” Trendelenburg sign. The kappa statistic calculated between the chiropractic practitioner and Vicon assessments for P_Drop_ and P_Disp_ were 0.22 (95% CI from − 0.12 to 0.55) and 0.25 (95% CI from − 0.09 to 0.59) respectively. These results demonstrate fair agreement between the practitioner and Vicon assessments [38].

## Discussion

This study investigated the relationship between hip abductor strength (measured as torque) and frontal plane pelvic motion during the Trendelenburg test. No significant correlations were found between the measures of peak hip abductor torque (isometric and isokinetic) and frontal plane pelvic motion (P_Drop_ and P_Disp_) recorded during the Trendelenburg test. These results show that the Trendelenburg test is not a valid assessment for hip abductor weakness. These findings also support previous work, in particular Kendall and colleagues, that found when hip abductor strength was decreased through a nerve block technique in a group of healthy male adult participants [18], and increased through hip abductor resistance training in a group of adult participants with chronic low-back pain [6], no significant changes to pelvic motion during the Trendelenburg test were found.

Although outliers in the data were identified, all data were retained in the primary analysis so as to preserve the clinical relevance of this study. This was done because the Trendelenburg test lacks the quantitative sensitivity to identify true outlier performance, as the test is reliant upon visual inspection and subjective decision making by the practitioner. Outlier removal can only be achieved by using objective data analysis techniques such as scatter plots or linear regression, which is beyond the scope of a practitioner in a clinical setting. In a secondary analysis, several outliers were removed from the original data sets because they were considered to be extreme and not truly representative of the population. After the removal of outliers, significant but weak relationships were found between (i) peak isometric torque and P_Disp_, (ii) peak isokinetic torque and P_Drop_, and, (iii) peak isokinetic torque and P_Disp_.

Reasons for the weak relationships found in this study may lie in the fact that the tests adopted were functionally different. Fundamentally, the mechanics of the tasks are different. During the Trendelenburg test, the hip abductors concentrically and isometrically contract to resist gravity to elevate and hold the pelvis respectively. For the isometric and isokinetic tests, the hip abduction motion is resisted by the lever arm of the Biodex. Furthermore, the Trendelenburg test requires submaximal isometric contraction of the stance side hip abductors so as to maintain maximal elevation of the pelvis over a period of 30 s [39]. On the other hand, the Biodex tests require maximal hip abduction effort (isometric and isokinetic muscle action) over a short time period of about 5 s. Hence, the role of the hip abductors during the Trendelenburg test and strength assessment tasks were very different. It can be argued that the Trendelenburg test requires submaximal isometric contraction of the hip abductors [39] combined with an endurance element to maintain the elevation of the pelvis whereas the Biodex tests required maximal hip abduction effort over a short time period. Studies have also shown that there is bilateral activation (electromyography) of the gluteus medius musculature during an isokinetic hip abduction test from a standing position [40] and unilateral activation on the stance limb side of the body during the Trendelenburg test [39]. These factors may partly explain the failure to find a relationship between the measures of peak hip abductor torque and pelvic motion during the Trendelenburg test since the demands on the hip abductor musculature are different. It is also important to note that the primary function of the hip abductor muscle group is to stabilise frontal plane motion of the pelvis during gait to prevent excessive pelvic drop or list (beyond 5°) on the swing limb side of the body [1–3]. This requires phasic submaximal contraction (concentric and isometric) of the hip abductors over a period of about 500 ms [41]. Hence the Trendelenburg test may have a stronger relationship with frontal plane pelvic motion during gait which is yet to be fully investigated.

A secondary aim of this study was to assess the clinical accuracy of the Trendelenburg test through direct comparison to 3-Dimensional motion analysis. Based upon the assessment by the practitioner, participants were classified as either “positive” or “negative”. Pelvic motion data from the respective groups were then compared using an independent t-test. No significant P_Drop_ difference was found between the groups but a significant mean group difference of 6 mm (*p* = 0.03) was found for the P_Disp_ data. While this shows statistical significance, this 6 mm may be too little of a difference for practitioners to observe. The examination of agreeance between the practitioner’s subjective assessment and the Vicon objective assessment found fair agreement for both the P_Drop_ (22% agreement) and P_Disp_ data (25% agreement), showing the P_Disp_ agreement to be slightly stronger. This suggests that vertical pelvic displacement data may be a better measure to assess pelvic motion during the Trendelenburg test. Moreover, this assessment can be easily performed with inexpensive 2-Dimensional motion analysis software. However, further work should be conducted with a larger sample size to confirm the use of this measure.

There were some limitations in this study. Firstly, during the Trendelenburg test, the Vicon motion capture required the practitioner to stand about three to four metres from the participant so as not to obstruct the view of a camera. This distance is further away than the one to two metres commonly used in a clinical setting and may have had some impact on their capacity to assess the test. A further limitation was only having one practitioner assess the Trendelenburg test. Therefore, the agreeance found in this may not be truly representative of the agreeance amongst the greater population of health practitioners. It is also not known if the MCID method used in this study was appropriate, as no other studies have used this statistic to investigate the Trendelenburg test. Future studies should also investigate the different methods or variations of the Trendelenburg test, involve greater participant numbers and clinical populations.

## Conclusion

In summary, this study found no significant relationship between normalised peak isometric and isokinetic hip abductor torque and frontal plane pelvic motion during the Trendelenburg test in a healthy young adult population. This suggests that the observed hip drop in the Trendelenburg test is not a valid test of weak hip abductor muscle strength, especially in comparison to the “gold standard” isokinetic dynamometry assessment. Weak but significant relationships were only found after outlier removal. There was also poor agreement between the practitioner and pelvic motion assessments indicating that an objectively measured hip drop is not always observable by the practitioner, giving rise to false positives and negatives. Recommendations for future studies are to investigate pathological populations, consider using the measurement of pelvic vertical displacement in “lieu of” or in addition to pelvic drop, and to consider investigating Trendelenburg test variations. While further study should be performed before any definitive conclusions can be made, the results of this study highlight that the Trendelenburg test may be inappropriate to identify hip abductor weakness in the absence of hip joint pathologies such as osteoarthritis.

## Data Availability

The datasets during and/or analysed during the current study available from the corresponding author on reasonable request.

## Abbreviations

ASIS: Anterior superior iliac spine
CI: Confidence interval
CSC: Clinically significant difference
IQR: Inter-quartile range
κ: Kappa statistic
MCID: Minimal clinically important difference
P_Drop_: Pelvic drop
P_Disp_: Pelvic vertical displacement
PSIS: Posterior superior iliac spine
